# How Is Health System Resilience Being Assessed? A Scoping Review

**DOI:** 10.34172/ijhpm.8097

**Published:** 2024-09-07

**Authors:** Calvin Tonga, Kristien Verdonck, Brice Essomba Edzoa, Olivia Ewokolo Ateba, Bruno Marchal, Joris Michielsen

**Affiliations:** ^1^Expanded Programme on Immunization, Ministry of Public Health, Yaoundé, Cameroon.; ^2^Department of Public Health, Institute of Tropical Medicine, Antwerp, Belgium.; ^3^Regional Delegation of Public Health for the Centre, Ministry of Public Health, Yaoundé, Cameroon.; ^4^Regional Delegation of Public Health for the South-West, Ministry of Public Health, Yaoundé, Cameroon.

**Keywords:** Scoping Review, Shock, Health System Resilience, System Mapping, Resilience Strategy, Resilience Capacity

## Abstract

**Background:**

It is commonly argued that resilient health systems ensure the well-being of populations even under critical conditions, whereas poorly resilient ones may be disrupted and collapse. We aimed to examine how health system resilience can be assessed as this issue is still under debate.

**Methods:**

We conducted a scoping review of peer-reviewed and grey literature published up to March 2022, following the Joanna Briggs Institute (JBI) guidance. CAIRN, DOAJ, E-Journals, Global Health Google Scholar, MedRxiv, OAIster, PubMed, reliefWeb, ScienceDirect, SmartResilience, SSRN, and World Health Organization (WHO) library were searched. The search strategy was based on key words from the research question and validated by an experienced librarian. We included full reports in English and French, whose primary focus was the health system, and that proposed or reported on the use of approaches for assessing health system resilience. Three independent reviewers did the selection and charting of reports. Extraction of information from the 34 reports that met the inclusion criteria followed predefined charting items.

**Results:**

Various definitions of the concept of health system resilience and diverging conceptual bases were found for the assessment of resilience, pointing at the lack of conceptual maturity. Three assessment approaches emerged from this review: (1) the system mapping approach which looks at health system core functions, (2) the capacity-based approach which focuses on the main characteristics of resilience, and (3) the strategy-based approach which examines resilience strategies. None of these approaches gives a full picture of resilience. They can be complementary; hence they are increasingly used in combination.

**Conclusion:**

This review identified three approaches to assessing health system resilience. The absence of a common understanding of what health system resilience represents still undermines its operationalisation and assessment. There is need for further testing and learning from empirical studies on the specific or integrated use of these frameworks.

## Background

 The COVID-19 pandemic has increased the focus on health system resilience, which initially emerged as a topic of interest in the field of health policy and systems research after the 2014 Ebola outbreak in West Africa.^1,2^ Mindful of the critique on the health system resilience discourse,^3,4^ we start from the definition of health system resilience by Kruk et al as “*the capacity of health actors, institutions, and populations to prepare for and effectively respond to crises; maintain core functions when a crisis hits; and, informed by lessons learned during the crisis, reorganise if conditions require it*.”^5^

 The concept of health system resilience emerged in the public health field less than two decades ago, and refers to how health systems respond to crises, shocks and stressors. Although popular in the global health discourse, there is still no common understanding of this concept.^6-9^ COVID-19 led to more questions about the ability of national health systems to cope with disruptive events, even in countries deemed to have high-performing health systems.^1,2,10^ Health systems are best considered as open complex adaptative systems, embedded within a specific context.^4^ Changes in the context can pose real challenges to health systems.^11,12^ This is reflected in the repeated calls from the World Health Organization (WHO) for improving the resilience of national health systems.^13,14^ It has been demonstrated that significant shifts in disease burden, natural disasters, economic or security crises among other events, can alter the performance of health systems and contribute to their disruption or collapse.^15^ The effects of the 2007 global financial crisis, the 2014 Ebola outbreak in West Africa and the current COVID-19 pandemic on even the best-performing health systems are illustrations of such challenges.^11^

 While the world is still struggling to control the COVID-19 pandemic and to deal with the consequences, we can anticipate that in the near future, health systems will most be hit by more shocks and crises as the result of the multiple effects of global warming, increasing population mobility, political unrest, and the consequences of war, as currently in Ukraine.^16–23^ Will health systems be responsive enough and maintain their core functions if faced by one or more disruptive events? Knowing the resilience status of a health system is crucial as only resilient health systems are in a position to provide optimal response in stress, shocks and crises.

 Building, improving or maintaining health system resilience begins with assessing the current resilience status. However, there is still debate about how this can be done and a validated set of indicators or an evidence-based framework for such assessments is still missing.^24-26^

 Previous reviews on health system resilience looked at the clarity and precision,^7,27^ as well as existing descriptions and frameworks for the concept.^9,28^ With this scoping review, we set out to identify and characterise existing approaches to assessing health system resilience. More specifically, we summarise current definitions and conceptual bases supporting the assessment of health system resilience, identify approaches for assessing health system resilience, and discuss the weaknesses and limitations of these approaches.

## Methods

###  Study Design

 Considering the complicated nature of the problem, the broad nature of the research objective, and the variety of literature on health system resilience assessment, we conducted a scoping review, following the five key stages recommended in the updated guidance proposed in the Joanna Briggs Institute (JBI) manual for evidence synthesis.^29^ Scoping reviews are relevant for mapping evidence on a topic, and identifying emerging themes, theories and sources, as well as knowledge or evidence gaps.^30,31^ The review protocol is available via the Figshare platform.^32^

###  Review Question

 The review question was developed following the Participants, Concept and Context framework,^31^ with no restriction of participants and context (Table 1). It was formulated as follows: *“According to the available literature, how can health system resilience be assessed?*”

**Table 1:** Details of the Framework Used for Developing the Research Question

**Domains**	**Elements**	**Description**
Participants – P	All people	No restriction in terms of population
Concept – C	Resilience	Resilience
	Assessment	Evaluation, appraisal or testing framework, tool, approach, strategy, metrics, measurement
Context – C	Health system	Components/building blocks or health system as a whole
	The world	No restriction in terms of geographical location, type of setting or cultural context

###  Inclusion Criteria

 Eligibility criteria were set in a way to ensure focus but remain inclusive and avoid potential omission of important information on the topic. They included the type of documents, language, publication dates and concepts of focus, as presented in Box 1.^33^ We searched for reports on studies that use, propose or discuss approaches, tools, methods, strategies or frameworks for assessing or measuring resilience. Only full document including peer-reviewed articles, reports, books, opinion papers and guidelines, written in English or French, and published between March 1, 2012 and February 28, 2022 were included in the review.

**Box 1. Eligibility Criteria**

**Inclusion Criteria**

- Full document written in English or French
- Peer-reviewed articles, reports, books, opinion papers, guidelines
- Published between March 1, 2012 and February 28, 2022
- Reflects on the topic, provides guidance or reports on the assessment of health system resilience
- Uses, proposes or discusses approaches, tools, methods, strategies or frameworks for assessing or measuring resilience
- Focuses on components or on the health system as a whole

**Exclusion Criteria**

- Focuses on individual psychological or ecological dimensions of resilience
- Focuses on other thematic area (eg, hospital organization, illness management, ecosystems)
- Conference proceedings, commentaries, letter to the editor, news articles, videos, and webpages
- Full text version not available
- Published before March 1, 2012

###  Search Strategy and Information Sources

 A search strategy to identify relevant documents in a systematic way was developed in consultation with an experienced librarian, based on key words from the research question. Peer-reviewed articles were searched in CAIRN, DOAJ, E-Journals, Global Health, Google Scholar, ITM Library Collection, PubMed, ScienceDirect, and SSRN. MedRxiv was searched for preprints whereas OAIster, reliefWeb, SmartResilience and WHO library were searched for grey literature (See Table S1, Supplementary file 1 for detailed search strategy). The database search was conducted from March 2-5, 2022. In addition, we applied a snowballing approach by reviewing reference lists of included documents to identify relevant documents that might have been missed in the electronic search.

###  Evidence Screening and Selection

 After duplicate identification and removal using the conditional formatting function and manual examination in Microsoft Excel, each document was screened on title and abstract, then on full text content. Three independent reviewers participated in the review process. Only records or reports validated by at least two reviewers were selected for the following step. All selected full texts were managed with Mendeley^®^ reference management software (version 2.66.0, Mendeley Ltd., 2022). We did not use formal tools to assess risk of bias in the selected papers, as this is not mandatory for scoping reviews.^31,34^

###  Data Charting

 A Microsoft Excel form for charting data from selected documents was developed in consultation among the three reviewers. To build a common understanding of its use and set up a systematic and reproducible data charting process, we conducted a pilot test during which all three reviewers did the charting of three reports together. Necessary adjustments were included in the final version of the form, allowing for independent charting of reports. Charting items included authors, country, objective, definition of resilience, reference framework, research design, study methods, shock or stressor, and weaknesses and limitations. Results from individual charting were merged and consensus was reached on the points of divergence during a meeting (See Table S9, Supplementary file 2 for detailed charting).

###  Data Collation Analysis

 The charted data were summarised in graphics (including charts and maps), narratives and tables. This was done in two steps. First, we developed a summary of the literature search, screening and inclusion process, and drafted a general description of the papers that were included in the review in terms of publication year, type of report, proposition or use of a conceptual basis, nature of the shock, country concerned. In a second step, we developed a synthesis organised in line with the specific objectives of the study, notably (1) the definitions and conceptual bases supporting the assessment of health system resilience, (2) the approaches used or proposed for assessing health system resilience, and (3) the weaknesses and limitations of identified approaches.

## Results

###  Search and Selection Process 

**Figure 1:**
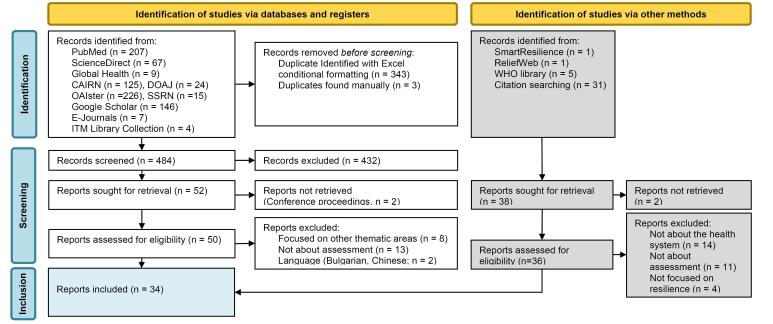
PRISMA Flow Diagram Presenting the Search and Selection of Reports Included in This Review. Abbreviations: PRISMA, Preferred Reporting Items for Systematic Reviews and Meta-Analyses; WHO, World Health Organization.

 The search strategy yielded 868 records. The electronic searches of online reference database yielded 830 records of which 346 duplicates were removed; 52 were selected based on their title and abstract, of which 27 met all inclusion criteria. Thirty-eight records were identified using the snowballing approach, of which 36 full texts were assessed; seven of them met all inclusion criteria. The process is summarised in the Preferred Reporting Items for Systematic Reviews and Meta-Analyses (PRISMA) flow diagram (Figure 1). A final set of 34 reports were included in the review.^24-26,28,35-64^

###  Characteristics of Included Reports

**Figure 2:**
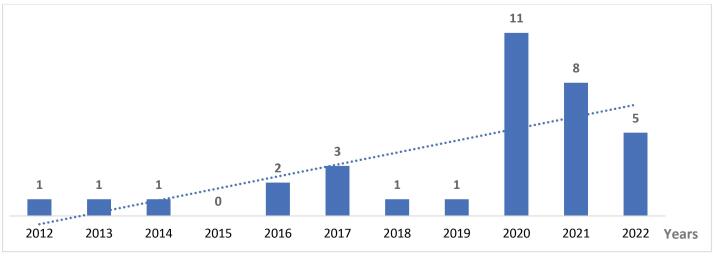
Distribution of Included Reports Over the Years.

 There are times of increased frequency of reports; from 2016 to 2017, then from 2020 to 2022 (Figure 2). These times correspond to the aftermath of a shock to health systems, notably the 2014-2015 Ebola virus disease outbreak in West Africa and the COVID-19 pandemic. Hence, the reports can be grouped in three periods: 2012-2015, 2016-2019 and 2020-2022.

 Twenty-one reports out of the 34 reports discuss case studies; 12/21 are about epidemiological shocks such as infectious disease outbreaks and pandemics (Table 2).

**Table 2:** Definitions of Resilience Provided in the Included Reports

**Source**	**Definition**	**View and Key Elements**	**References**
Ammar et al, 2016	Capacity of a health system to absorb internal or external shocks (for example prevent or contain disease outbreaks) and maintain functional health institutions while sustaining achievements	*Capacity*: Absorb, maintain, sustain	NA^a^
Bayntun et al, 2012	Capability of the public health and health-care systems, communities, and individuals to prevent, protect against, quickly respond to, and recover from health emergencies, particularly those whose scale, timing, or unpredictability threatens to overwhelm routine capabilities	*Capability*: Prevent, protect, respond, recover	Nelson et al, 2007
Bhandari and Alonge, 2020	Process linking a set of networked adaptive capacities (resources with their dynamic attributes) at individual or community level to a positive trajectory of functioning and adaptation of the health system at the community level after a health shock	*Process*: Adapt, Maintain	Norris et al, 2008
Bigoni et al, 2022	Capacity to absorb the impacts of external shocks caused by epidemics, natural disasters, economic crises, or other causes without altering its operations and avoiding an increase of unmet health needs for different reasons	*Capacity*: Absorb, maintain, sustain	Thomas et al, 2020
Crowe et al, 2014	Capability of a health system to mitigate the impact of major external disruptions on its ability to meet the needs of the population during the disruption	*Capacity*: Mitigate, sustain	NA
Etemadi and Tadayon, 2021	Capacity to prepare and respond effectively to crises, while maintaining the key functions of the health system before, during and after the crisis	*Capacity*: Prepare, respond, maintain	Nuzzo et al, 2019
Expert Group HSPA, 2020	Capacity of a health system to (*a*) proactively foresee; (*b*) absorb; and (*c*) adapt to shocks and structural changes in a way that allows it to (*i*) sustain required operations; (*ii*) resume optimal performance as quickly as possible; (*iii*) transform its structure and functions to strengthen the system; and (possibly) (*iv*) reduce its vulnerability to similar shocks and structural changes in the future	*Capacity: *Foresee, absorb, adapt, maintain, resume, transform, reduce vulnerability	NA
Foroughi et al, 2022	Ability of the system to prepare for and respond to sudden shocks and everyday challenges and its capacity to absorb deteriorations, adapt, and transform the health system to cope with them	*Ability*: Prepare, Respond *Capacity*: Absorb, adapt, transform	Thomas et al, 2020; Barasa et al, 2018
Giarelli, 2020	Capacity to absorb, adapt and transform when exposed to a shock such as a pandemic, natural disaster, armed conflict or a financial crisis and still retain the same control over its structure and functions	*Capacity*: Absorb, adapt, transform, maintain	Blanchet et al, 2017
Gilson et al, 2020	Characteristic of complex, adaptive health systems that allows them to respond to chronic stress in ways that transform how they function	*Characteristic*: Respond, transform	Barasa et al, 2017
Haldane et al, 2021	Institutions’ and health actors’ capacities to prepare for, recover from and absorb shocks, while maintaining core functions and serving the ongoing and acute care needs of their communities. During a crisis, a resilient health system is able to effectively adapt in response to dynamic situations and reduce vulnerability across and beyond the system	*Capacity*: Prepare, respond, maintain, re-organise	Kruk et al, 2015
Jovanović et al, 2020	Ability to understand and anticipate the risks - including new/emerging risks -threatening the critical functionality of the infrastructure, prepare for anticipated or unexpected disruptive events, optimally absorb/withstand their impacts, respond and recover from them, and adapt/transform the infrastructure or its operation based on lessons learned	*Ability*: Understand, anticipate/prepare, absorb/withstand, respond/recover, adapt/transform	Russoa and Ciancarinia, 2016
Kagwanja et al, 2020	Maintenance of positive adjustment under challenging conditions such that the organisation emerges from those conditions strengthened and more resourceful	*Capacity*: Maintain, emerge	Gilson et al, 2017
Karamagi et al, 2022	Capacity to “prepare and effectively respond to crises; maintain core functions; and, informed by lessons learnt, reorganize if conditions require it”	*Capacity*: Prepare, respond, maintain, learn, re-organise	Kruk et al, 2015
Kruk et al, 2017	Capacity of health actors, institutions, and populations to prepare for and effectively respond to crises; maintain core functions when a crisis hits; and, informed by lessons learnt during the crisis, re-organise if conditions require it	*Capacity*: Prepare, respond, maintain, learn, re-organise	Kruk et al, 2015
Ling et al, 2017	Capacity to prepare for and effectively respond to crises while maintaining core health system functions pre-, during, and post-crisis	*Capacity*: Prepare, respond, maintain	Kruk et al, 2015
Lo Sardo et al, 2019	Resilience quantifies the rate of recovery and the extent to which a system is able to recover from disruptive events	*Ability*: Recover	Woods, 2015
Massuda et al, 2021	Capacity of health agents, institutions, and populations to prepare themselves to respond to such shocks, keeping the systems’ essential functions without changing health outcomes, as well as the ability to reorganize from lessons learned	*Capacity*: Prepare, respond, maintain, re-organise, learn	Kruk et al, 2017
McKenzie et al, 2016	Capacity of a health system to deal with change, to adapt and transform, and to maintain relevance, when confronted by such major disruptions	*Capacity*: Deal with, adapt, transform, maintain	Kruk et al, 2015
Meyer et al, 2020	Capacities that could potentially strengthen health system to either infectious disease threats or natural hazards	*Capacity*	Kruk et al, 2015
Ozen and Tuncay, 2021	Capacity of health actors, institutions, and populations to prepare for and effectively respond to crises; maintain core functions when a crisis hits; and, informed by lessons learnt during the crisis, and re-organise if conditions require it	*Capacity*: Prepare, respond, maintain, learn, re-organise	Kruk et al, 2015
Pilevari and Shiva, 2021	Providing the community with the best available and equitable care, withstand shocks, endure crisis and support people against hardships and uncertainty of all kinds when national health integrity is at risk	Maintain, Withstand, endure crisis, support people	NA
Rios et al, 2020	Capability of a health system to prepare, respond and reorganize under conditions of stress, is posited to protect the population from excess morbidity and mortality	*Capability*: Prepare, respond, re-organise	Kruk et al, 2015
Rogers et al, 2021	Capacity of a health system to (*a*) proactively foresee; (*b*) absorb; and (*c*) adapt to shocks and structural changes in a way that allows it to (*i*) sustain required operations; (*ii*) resume optimal performance as quickly as possible; (*iii*) transform its structure and functions to strengthen the system; and (possibly) (iv) reduce its vulnerability to similar shocks and structural changes in the future	*Capacity:* Foresee, absorb, adapt, maintain; resume, transform, reduce vulnerability	Expert Group HSPA, 2020
Thomas et al, 2013	Capacity of a system to absorb disturbance and reorganise while undergoing change so as to still retain essentially the same function, structure, identity and feedback	*Capacity*: Absorb, re-organise, maintain	Walker et al, 2004
Thomas et al, 2020	Ability to prepare for, manage (absorb, adapt and transform) and learn from shocks	*Ability*: Prepare, absorb, adapt, transform, learn	NA
Wang et al, 2020	Capacity to effectively prepare for and respond to pandemics while maintaining core functions, informed by lessons learned on an ongoing basis, and reorganize promptly if conditions require it	*Capacity*: Prepare, respond, maintain, learn, re-organise	Kruk et al, 2015
WHO Regional Office for Africa, 2018	Inbuilt capacity of the system to sustain provision of essential health and health-related services even when challenged by outbreaks, disasters, or other shocks	*Capacity*: Maintain	NA
WHO, 2022	Capability of anticipating, responding to, coping with, recovering from, and adapting to climate-related shocks and stresses, so as to bring about sustained improvements in population health, despite an unstable climate	*Capability*: Anticipate, respond, cope, recover, adapt	Kruk et al, 2015; Thomas et al, 2020

Abbreviations: HSPA, Health Systems Performance Assessment; WHO, World Health Organization. ^a^No reference cited for the definition.

**Figure 3:**
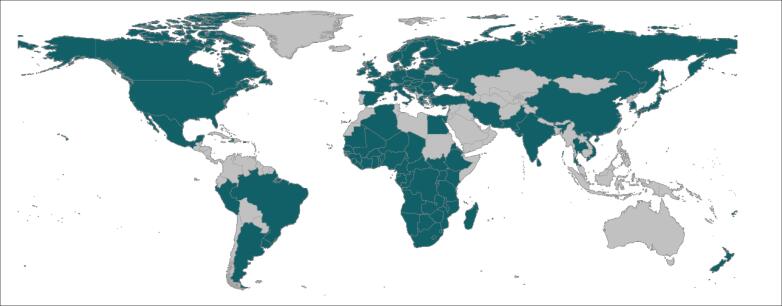
Countries’ Health System Resilience Assessment Map (Darker Areas).

 Thirteen out of the 34 reports propose an approach for assessing health system resilience; 16/34 apply such an approach; and the remaining 5/34 both propose and apply an approach to assess health system resilience. The reports describe assessments of health systems resilience in 103 countries (Figure 3; see Table S2, Supplementary file 1 for detailed list of countries).

###  Definitions of Health System Resilience

 Definitions of health system resilience were found in 29 (85%) reports (Table 2). Six reports used a definition without reference. Twenty-four references were cited for definitions, mostly Kruk et al,^5^ (cited 11/24; 46%), followed by Thomas et al,^26^ who were cited 3 times (13%). We identified 25 key terms from the analysis of definitions for a total number of 106 occurrence (See Table S3, Supplementary file 1 for the full list of key terms). The most frequent were: “maintain” (19/106; 18%), “respond” (14/106; 13%), “prepare” (12/106; 11%), “absorb” (9/106; 8%), “adapt” (9/106; 8%), “transform” (8/106, 8%) and “re-organise” (7/106; 7%).

 The definition of resilience has evolved with time, incorporating new elements and living out or modifying others. “*Adapt,”* “*prepare,”* “*transform,”* and “*learn” *were introduced mainly during the second period, between 2016 and 2019, in line with the definition by Kruk et al.^5,24,48^ In the third period, from 2020 to 2022, “*anticipate,”*^42,58^ “*foresee,”*^37,55^ and “*support people”*^52^ were introduced. On the other hand, “*prevent” *and* “protect”* did no longer appear since 2015; this may illustrate some distancing from the emergency preparedness and response field by health system resilience scholars (Table 3). The second period is thus marked by the emergence of the transformative dimension of resilience, which is maintained in the third period, where an additional emphasis is placed on vulnerability reduction (See Tables S4, Supplementary file 1 for the evolution of key references).

**Table 3:** Evolution of Key Elements Used in Defining Resilience

**Periods**	2012-2015		2016-2019		2020-2022	
**Number of reports**	3		6		20	
**Key elements from** **definitions with** **their percentage of** **occurrence**	Elements^a^	%	Elements	%	Elements	%
	*Absorb*	11.1%	*Absorb*	5.9%	*Absorb*	8.6%
	*Maintain*	11.1%	*Maintain*	29.4%	*Maintain*	16.0%
	*Mitigate*	11.1%	*Deal with*	5.9%	*Cope/withstand/endure*	4.9%
	Prevent	11.1%				
	Protect	11.1%				
	*Recover*	11.1%	*Recover*	5.9%	*Recover/resume/emerge*	6.2%
	*Re-organise*	11.1%	*Re-organise*	5.9%	*Re-organise*	7.4%
	*Respond*	11.1%	*Respond*	11.8%	*Respond*	13.6%
	*Sustain*	11.1%	*Sustain*	5.9%	*Sustain*	1.2%
			**Adapt**	5.9%	*Adapt*	9.9%
			**Learn**	5.9%	*Learn/understand*	2.5%
			**Prepare**	5.9%	*Prepare/ Reduce vulnerability*	14.8%
			**Transform**	5.9%	*Transform*	8.6%
					**Anticipate/foresee**	4.9%
					**Support people**	1.2%

^a^ Key elements that are maintained across the periods are *italicised*; new elements are in bold.

 Most authors (27; 93%) viewed resilience as an *ability* (ie, an inherent competency)*, a capacity (*ie, a faculty that is displayed), *or a capability* (ie, a faculty or process that can be developed). By contrast, Bhandari and Alongepresented resilience as a *process*, and Gilson et al considered it to be a *characteristic* of complex adaptive systems.^40,62^

###  Conceptual Frameworks for Assessing Health System Resilience

 Twenty-four different conceptual bases were identified, of which four were found more than once:

- The resilient health system framework from Kruk et al,^5^ found in six reports;
- The health system building blocks framework from the WHO,^66^ found in five reports;
- The everyday health system resilience framework from Kagwanja et al,^43^ found in two reports;
- The 13 resilience-enhancing strategies from the Expert Group on Health Systems Performance Assessment,^37^ also found in two reports.

The diversity of conceptual frameworks increased from three between 2012 and 2015, to five in 2016-2019, and 19 in 2020-2022. The health system building blocks framework^66^ has continuously been used over the last decade (See Table S5, Supplementary file 2 for the chronology of conceptual bases).

###  Approaches for Assessing Health System Resilience 

 In line with previous research by Turenne et al, Rohova and Koeva, and Foroughi et al, we classified approaches to assess health system resilience as “system mapping,” “capacity-based,” and “strategy-based.”^7,27,28^ “System mapping approach” aims to capture resilience through the assessment of the organisation and/or performance of core functions of the health system. It may focus on one or more functions, and go beyond the traditional six building blocks,^66^ to include other components as values or community participation, depending on the health system framework that is used to guide the process.^11,67^ “Capacity-based approach” captures resilience through the assessments of system capacities or characteristics that facilitate resilience; it builds primarily on the resilient health system framework by Kruk et al.^5^ This approach identifies and describes elements attesting to the *awareness, diversity, self-regulation, integration *and* adaptability* of the system, as well as system gaps. “Strategy-based approach” describes how a shock affects the system and what mechanisms are developed as part of the *absorptive*, *adaptive*, and *transformative* strategies.^43,68^

 The diversity and frequency of approaches varied across the periods (Table 4; see Table S6, Supplementary file 1 for detailed list of assessment approaches per author). The “capacity-based” approach emerged in the 2016-2019 period. Six reports (6/34; 18%), which are grounded in the Kruk et al conceptual framework (five dimensions of resilience), used or propose this approach.^5^ The researchers using this approach assessed or described resilience through the five elements of the framework, identifying and describing system gaps.

**Table 4:** Proposed/Used Assessment Approaches

**Assessment Approaches**	**Period**			
	**2012-2015**	**2016-2019**	**2020-2022**	**Total**
System mapping approach	2	4	14	20
Strategy-based approach	1	0	4	5
Capacity-based approach	0	3	3	6
Mix of approaches	0	0	3	3
**Total**	**3**	**7**	**24**	**34**

 The “strategy-based” approach was proposed or used in five reports (5/34; 15%) to assess the *absorptive*, *adaptive* and *transformative* strategies developed by the health system. Authors have explored the health system pre-requisites to a strategy-based approach, including funding, provision (service delivery and availability of resource such as workforce, medical products, vaccines and technologies), and governance. Governance is further examined in terms of managerial characteristics. These include knowledge, legitimacy, uncertainties, and interdependence capacities for studies drawing on the Blanchet et alframework,^68^ and cognitive, behavioural and contextual capacities for studies grounded on the everyday health system resilience framework.^43^

 The “system mapping” approach has continuously been referred to and remains the dominant approach in the 2020-2022 period. This approach was used or proposed in 20 reports for assessing health system resilience (20/34; 59%), typically focusing on core functions, outputs and outcomes of the health system.

 Since 2021, a number of papers have proposed or reported the use of a combination of approaches for assessing health system resilience (3/34; 9%).^28,44,52^ Assessment approaches are, indeed, not mutually exclusive. Karamagi et al give an example of mix of approaches for assessing health system resilience.^44^ They generated a combined health system resilience index by associating an inherent system resilience index with an emergency preparedness and response index. The inherent system resilience index is grounded in the Kruk et al framework,^5^ thus assessing resilience using the capacity-based approach, whereas the emergency preparedness and response index is grounded in the 2005 International Health Regulation, thus uses the system mapping approach.

 In terms of methods, qualitative and mixed methods were used with the three assessment approaches. Evidence review and exclusive use of quantitative methods were only found with the system mapping approach (Table 5; see Table S7, Supplementary file 1 for more details). We also checked whether assessments were carried out during or after the shock under study. Four on six studies (67%) using or proposing a capacity-based approach were conducted in the absence of a shock, whereas all five studies (100%) using a strategy-based approach were conducted during the shock. Four on twenty studies (25%) using or proposing a system mapping approach were conducted in the absence of a shock and nine (45%) during a shock. The three approaches are used at country level as well as at lower levels of national health systems (See Table S8, Supplementary file 1 for level of use).

**Table 5:** Characteristics and Focus of Studies With Each of the Assessment Approaches

**Assessment Approach**	**Associated Conceptual Basis**	**Study Methods**^a^				**Assessed**		**Type of Results**
		**Evidence Synthesis**	**Quantitative**	**Qualitative**	**Mixed**	**Components/Capacities/Dimensions**	**Frequency**	
System mapping	(i) Briguglio’s vulnerability and resilience framework (ii) CDC’s EPHS framework (iii) Complex adaptive systems theory (iv) Conceptual framework for EID preparedness (v) Determinants of resilient health systems framework (vi) Health system resilience index (vii) Health system building blocks (viii) Input-output-outcome (ix) Production process (x) Stylised health system, akin to an industrial process	3 (25%)	8 (66.7%)	2 (16.7%)	2 (16.7%)	Financing	6 (46.2%)	Metrics and narrative
						Infrastructures	5 (38.5%)	
						Health workforce	9 (69.2%)	
						Information systems	9 (69.2%)	
						Leadership and governance	8 (61.5%)	
						Engagement with communities and other sectors	8 (61.5%)	
						Service delivery	6 (46.2%)	
						Access to healthcare	3 (23.1%)	
						Equity	2 (15.4%)	
						Health outcome	1 (7.7%)	
						Medical/non-medical products and technologies	5 (38.5%)	
Strategy-based approach	(i) Conceptual framework of the dimensions of resilience governance (ii) Everyday HS resilience framework (iii) Framework for assessing how health systems adjusted to economic crisis (iv) HS resilience analytical framework	0 (0%)	0 (0%)	1 (25%)	3 (75%)	Absorption	5 (100%)	Narrative
						Adaptation	5 (100%)	
						Transformation	5 (100%)	
						Health system pre-requisites (Funding, provision, governance^b^)	4 (80%)	
Capacity-based approach	(i) Health system resilience index (ii) Resilient health systems framework	0 (0%)	0 (0%)	3 (60%)	2 (40%)	Awareness	5 (100%)	Narrative
						Diversity	5 (100%)	
						Integration/mobilisation	5 (100%)	
						Adaptability and learning/transformation	5 (100%)	
						Self-regulation	5 (100%)	

Abbreviations: CDC, Centers for Disease Control and Prevention; EPHS, Essential Public Health Services; EID, emerging infectious diseases; HS, health system. ^a^Method could not be determined for one report. ^b^Governance included knowledge, legitimacy, uncertainties, interdependence capacities,^68^ or cognitive, behavioural and contextual capacities.^43^

###  Metrics Used for Assessing Health System Resilience

 Metrics were found only in reports using the system mapping approach. These metrics are presented in Table 6; they consist of health system input, output and outcome indicators. However, no metrics were found for assessing governance and other “soft” components like values or trust.

**Table 6:** Metrics Used for System Mapping

**Health System Indicators’ Categories**		**Metrics**
Inputs	Health workforce^53,59,60,63^	Figures and trends of (*i*) physicians, nurses, nurse-aids and other professional categories, (*ii*) training, safety and protection activities, and (*iii*) Incentives
	Infrastructure^35,59,60^	Figures and trends of (*i*) functional health facilities, (*ii*) hospital beds, and (*iii*) quantity of available vs required
	Information/surveillance & monitoring systems^59^	Data completeness Data quality and access
	Medical/non-medical products and technologies^35,59^	Figures and trends of equipment and drugs available vs required
	Financing^51,60^	Figures and trends of (*i*) state budget allocated to the health sector, (*ii*) funding from donors, (*iii*) funds transferred to lower levels, (*iv*) financial protection including subsidisation of healthcare and insurance, (*v*) payment delays, and (*vi*) efficiency of health expenditures
Outputs	Service provision and equity^35,60,63^	Figures and trends of (*i*) functional health programmes, (*ii*) level of implementation of planned activities, (*ii*) activities targeting vulnerable/hard-to-reach groups, (*iii*) outbreak response campaigns, (*iv*) quantity of healthcare provided (childbirth, screening, physician appointment, surgeries, other procedures), and (*v*) patient satisfaction
Outcome	Utilisation of healthcare^19,50,60,63^	Figures and trends of (*i*) unmet demand, and (*ii*) coverage of interventions, including mass activities (campaigns), (*iii*) treatment success rate, and (*iv*) disease outbreaks/case new diseases
Impact	^19,50,51,60^	Figures and trends of (*i*) morbidity of selected diseases, (*ii*) infant and under 5 mortality, (*iii*) maternal mortality, (*iv*) excess death due to specific disease, and (*v*) out-of-pocket expenditures

## Discussion

 Our review confirms that a diversity of definitions of health system resilience are being used. Definitions have evolved with time, whereby authors often integrate some new terms and views, while leaving or rephrasing others. Authors tend to use and modify definitions based on gaps they identify, the purpose of their specific study and their own interpretation of the concept. This variety of definitions is observed even in the recent literature and attests to the lack of maturity of the concept of health systems resilience, confirming the results of Turenne et al^7^ There are also still inconsistencies in the use of terms across frameworks. While Blanchet et al consider absorption, adaptation and transformation as “capacities,”^68^ Kagwanja et al label these as “strategies.”^43^ What the latter consider to be “management capacities” are “dimensions” for the first. Moreso, Kruk et al referred to components of their framework as elements that “*characterise a resilient health system,*” or “*characteristics of resilience,*”^5^ not as dimensions as extensively presented in the literature. The word ‘dimension’ as used by Kruk et al would be better understood as ‘attributes,” and “dimensions” in the Blanchet et al framework as “strategies.”

 It should be noted that a concept reaches maturity when it has a consensual definition, clear characteristics, defined limits and meets some essential preconditions.^7^ This presents a major challenge to Health Systems and Policy Research scholars as conceptual maturity is a key requirement for effective operationalisation of concepts and of assessment methods. It should be noted that efforts are made to come to a more comprehensive definition, as reported by Rogers et al who define health system resilience as “*the capacity of a health system to (a) proactively foresee; (b) absorb; and (c) adapt to shocks and structural changes in a way that allows it to ( i ) sustain required operations; (ii) resume optimal performance as quickly as possible; (iii) transform its structure and functions to strengthen the system; and (possibly) (iv) reduce its vulnerability to similar shocks and structural changes in the future*.” This definition was developed by the Expert Group on Health Systems Performance Assessment,^37^ and used for assessing the Resilience of Health Systems in Europe.

 We found that 24 conceptual frameworks have been used in the reviewed reports to assess health system resilience, drawn from various disciplines, including public health, ecology, social sciences, security studies, and emergencies. Only two conceptual frameworks were referred to more than 5 times: the resilient health system framework,^5^ and the WHO health system building blocks.^66^ It should be noted that the latter is in essence a simple frame designed for discussing health system strengthening, not resilience. The variety of conceptual bases reflects the inconsistency in the definition of health system resilience. Authors develop, adopt or/and adjust a framework according to their understanding of the concept, the gaps they identify or the purpose of their specific study, which further illustrates the lack of clarity of the meaning of health system resilience.^3,6,7^

 It struck us that only a few authors refer to frameworks from other disciplines, although efforts towards the development and operationalisation of the concept of resilience originally began in fields like ecology and natural resource management. We found that more recently, relevant work has been done in the fields of disaster management, food security and economics among others. Many international development organisations have developed resilience assessment models, including the United Nations Development Programme, the Food and Agriculture Organisation and the United States Agency for International Development.^69^ Their work could enrich ongoing efforts for assessing health systems resilience.

 Among the papers we reviewed, we identified three approaches to assessing health system resilience: (*i*) the *system mapping approach*, (*ii*) the *strategy-based approach*, and (*iii*) the *capacity-based* approach.^7,27,28^

 The *system mapping approach* builds primarily on the WHO six building blocks framework^66^ and is the most used. It is a health system performance assessment frame more than a resilience assessment framework, as can be confirmed from the metrics it uses to assess the six functions of a health system. We assume its popularity derives from its easy fit with an input-output-outcome logic, often used in the assessment of public health interventions, projects and programmes. Proponents of the six building block framework argue that it can be used at any phase of the shock cycle that includes (*i*) the pre-shock stage, the (*ii*) shock onset stage, (*iii*) the shock impact stage, and (*iv*) the post-shock stage.^37^ As a further advantage, authors mention that conventional surveys and administrative reports as well as routinely collected data can be used to assess resilience with this approach.^25,44,56^ This would allow for a rapid assessment at a relatively low cost, despite some concerns with the quality of routine data. It would also facilitate standardisation and comparison, which in turn would allow the identification and prioritisation of settings requiring urgent action. Although it is the most used approach, system mapping allows only for an *indirect assessment* of health system resilience as it considers only observable or measurable “effects” of interventions on core functions of the health system. Importantly, this approach has inherited the shortcomings of the framework: it is linear and static, and blind to the underlying mechanisms of both shock and resilience. Indeed, it ignores the complexity and dynamics inherent to the health system as it does not capture the complex interactions between the various components, nor the role and opinion of the people for whom a resilient health system is supposedly built. It should be noted that in response to the current debate on health system frameworks, some authors added new components to their assessment framework, including community engagement.^41,67^

 The *capacity-based approach* builds primarily on the resilient health system framework by Kruk et al.^5^ The researchers using the capacity-based approach assess resilience through the five elements of the framework, namely *awareness*, *diversity*, *self-regulation*, *integration,* and *adaptability* of the system. It is mostly used in studies carried out before or after a shock. Kruk and colleagues refer to the Rockefeller’s City Resilience Framework as a source of inspiration and ‘tested’ their frame in three case studies but, here too, the theoretical underpinnings of the choice of the five elements are not well developed.

 The *strategy-based approach *builds primarily on the absorptive, adaptive, and transformative strategies developed by actors in resilient health systems. Authors using this approach describe how the shock impacts the system and what mechanisms are developed under each of the three strategies. They describe each strategy, which is considered a process for dealing with issues created by the shock. This approach has recently been adapted by Blanchet et al in their framework on the dimensions of resilience governance, and by Kagwanja et al in their everyday health system resilience framework.^43,68^ Grounded in complex systems theory, Blanchet et al included a governance component to the framework, with four interlinked management capacities, including knowledge management, management of uncertainties, the capacity to manage interdependence and the capacity to build or develop legitimacy.^68^ Kagwanja et al included three health system resilience capacities, namely cognitive, behavioural and contextual capacities, which are *in se* strategic management capacities.^43^ These adjustments are illustrative of the perceived need to identify precursors or determinants of resilience: the focus is set on capacities required to better manage resilience, prior to the shock. Proponents argue that assessments of health system resilience using this approach provide details about mechanisms for resilience, with a broad view on interactions and the complex nature of any response to a shock. This approach is mostly used in studies during the shock and requires the assessment team to be embedded in the system, mapping and describing the shock and resilience processes. It may require long-term studies to assess resilience to structural challenges. Critique on this approach includes the charge that it is simply “change management.” However, it could be argued that if ensuring resilience is about addressing structural factors that weaken a health system and indeed a society, this is half a management responsibility and half a society’s responsibility. Recent adjustments to the primary framework show that its conceptualisation is still to reach full maturity.

 Both the capacity-based and strategy-based approaches look directly into how resilience is developed and unfolds when the health system is faced with a shock. They may be considered approaches to *direct assessment *of health system resilience. However, we argue that resilience does not emerge from a vacuum; it is an emerging feature of a health system. Describing processes labelled as “resilience” makes little sense if the link between these processes and the aim of a health system is not assessed, which is to protect human life and achieve positive health outcomes for all, in everyday functioning as well as during and after a shock.^5^ Such processes are conducted by the actors and with resources of the system. For example, medicines must be available before rationing can be implemented as an absorptive strategy; governance, implementation of change strategies and other activities, are driven by actors who are part of the system, whereas knowledge and awareness are mostly built from the output of the information system. Many authors using these approaches are aware of this; they therefore often mention pre-requisites to resilience that usually correspond to the resources of the health system. Moreso, to complement their description and give “tangible” evidence of resilience, they report on health system inputs, outputs and/or outcomes, similar to the system mapping approach.^40,43,56,64^

 None of the three approaches seems to pay much attention to the structural political, social, economic, and other determinants of health system performance (or the lack thereof). They do not tackle the structural disturbances weakening health systems, which has been raised as a main issue by other authors.^3^

 It clearly appears that none of the approaches gives a full picture of resilience, nor that any is adapted to all contexts, shock types and phases of the shock cycle. They are also not mutually exclusive, but complementary. Some researchers are proposing assessment frameworks using a mix of approaches, and this may be the start of a new trend.^28,44^ Foroughi et al, for instance,noted that each of the major health system resilience frameworks focuses on one or two of the aspects necessary for the operationalisation of this concept.^28^ The authors developed a frame that combines their core elements into one comprehensive framework, centred on the six building blocks framework. However, authors of these integrated approaches do not explicitly develop theoretical foundations. Furthermore, there is still a need for further testing and learning from the field on their specific use.

 Better definitions and frames are needed before comprehensive sets of indicators can be proposed. Also, assessments metrics should be customized to the level within the health system, the type of shock and the phase of resilience.

 This review has some limitations. We may have missed some papers during the search. The full texts of two records could not be retrieved although we contacted the authors for this purpose. Also, our inclusion criteria only considered full-text reports published before 01/03/2012 in English and French; yet, our search found some records with full texts in Chinese and Czech languages.

## Conclusions

 Although there has been a growing interest in the concept of resilience over the past decade, there is still no consensus on its definition, nor a validated approach for assessing health system resilience. This clearly owes to changes and diversity in the understanding of health system resilience, which is gradually evolving to incorporate criticism and contributions from various fields of research and practice. Three main assessment approaches emerged from this review: the system mapping which looks at the health system core functions, the capacity-based which focuses on the main characteristics of resilience, and the strategy-based which examines resilience strategies. None of these approaches gives a full picture of resilience. They are not mutually exclusive and can be complementary. The absence of a common understanding of the concept of health system resilience represents a major hinderance to its operationalisation and assessment. We therefore suggest the following priority areas as a way forward for the Health Systems and Policy Research community:

- To further research into the factors that shape the resilience of a health system, whereby cross-fertilisation between fields like individual resilience, community resilience, resilience of social protection and health financing system and urban resilience is explored;
- To further test the current assessment approaches, separately or in combination;
- To further explore how the type of shock and the phases of a shock combine with pre-existing capacities to shape the resilience of a system;
- To build and test a theory on health system resilience.

## Ethical issues

 Not applicable.

## Conflict of interests

 Authors declare that they have no conflict of interests.

## Supplementary files


Supplementary file 1. Detailed Search Strategy and Analyses.

Supplementary file 2. Data Charting.
