# Supplementary material for: How Is Health System Resilience Being Assessed? A Scoping Review
Source: Int J Health Policy Manag. 2024 Sep 7;13:8097. doi: 10.34172/ijhpm.8097 (PMC11496737; doi:10.34172/ijhpm.8097)
Supplement: Supplementary file 2 — Data Charting. [file ijhpm-13-8097-s002.pdf]

**Article title:** How Is Health System Resilience Being Assessed? A Scoping Review

**Journal name:** International Journal of Health Policy and Management (IJHPM)

**Authors' information:** Calvin Tonga[1]*, Kristien Verdonck[2], Brice Essomba Edzoa[3], Olivia Ewokolo Ateba[4], Bruno Marchal[2], Joris Michielsen[2]

[1]Expanded Programme on Immunization, Ministry of Public Health, Yaoundé, Cameroon.

[2]Department of Public Health, Institute of Tropical Medicine, Antwerp, Belgium.

[3]Regional Delegation of Public Health for the Centre, Ministry of Public Health, Yaoundé, Cameroon.

[4]Regional Delegation of Public Health for the SouthWest, Ministry of Public Health, Yaoundé, Cameroon

***Correspondence to:** Calvin Tonga; Email: clvntonga3@gmail.com

**Citation:** Tonga C, Verdonck K, Edzoa BE, Ateba OE, Marchal B, Michielsen J. How is health system resilience being assessed? A scoping review. Int J Health Policy Manag. 2024;13:8097. doi:10.34172/ijhpm.8097

**Supplementary file 2.** Data Charting

Table S2: Detailed search strategy

| Source | Type of literature | Search terms |
|---|---|---|
| PubMed | Peer reviewed | ("health system"[Title/Abstract] OR "health systems"[Title/Abstract] OR "healthcare system"[Title/Abstract] OR "healthcare systems"[Title/Abstract] OR "healthcare system"[Title/Abstract] OR "health care systems"[Title/Abstract]) AND ("resilience"[Title/Abstract] OR "resiliences"[Title/Abstract] OR "resiliencies"[Title/Abstract] OR "resiliency"[Title/Abstract] OR "resilient"[Title/Abstract] OR "resilients"[Title/Abstract]) AND ("assess"[Title/Abstract] OR "assessed"[Title/Abstract] OR "assessment"[Title/Abstract] OR "assesses"[Title/Abstract] OR "assessing"[Title/Abstract] OR "assessment"[Title/Abstract] OR "assessment s"[Title/Abstract] OR "assessments"[Title/Abstract] OR "appraised"[Title/Abstract] OR "appraisal"[Title/Abstract] OR "appraising"[Title/Abstract] OR "evaluated"[Title/Abstract] OR "evaluation"[Title/Abstract] OR "evaluating"[Title/Abstract]) AND ("framework"[Title/Abstract] OR "framework s"[Title/Abstract] OR "frameworks"[Title/Abstract] OR ("tool"[Title/Abstract] OR "tools"[Title/Abstract] OR "tool s"[Title/Abstract]) OR ("approach"[Title/Abstract] OR "approaches"[Title/Abstract]) OR ("strategy"[Title/Abstract] OR "strategies"[Title/Abstract]) OR ("metrics"[Title/Abstract] OR "metric"[Title/Abstract]) OR ("measure"[Title/Abstract] OR "measuring"[Title/Abstract] OR "measurement"[Title/Abstract])) AND (y_10[Filter]) |
| Science Direct | Peer reviewed | health system AND resilience AND (assessment OR appraisal OR evaluation OR test) AND (tool OR approach); Title, abstract or author-specified keywords; *2012-2022* |
| | | health system AND resilience AND (assessment OR appraisal OR evaluation OR test) AND (framework OR method); Title, abstract or author-specified keywords; *2012-2022* |

| Source | Type of literature | Search terms |
|---|---|---|
| | | health system AND resilience AND (assessing OR appraising OR evaluating OR testing) AND (framework OR method); Title, abstract or author-specified keywords; *2012-2022* |
| EBSCOh ost | Peer reviewed | "Heath system" resilience assessment framework; *find all my search terms, apply related words, year of publication: 20120301-20220228, ITM All collection, all languages, all results, all databases* |
| CAIRN | Peer reviewed | "health system" AND (resilience OR sustainability) AND (assessment OR appraisal OR evaluation OR test) AND (tool OR approach); *intégral, 10 dernières années, revues, santé publique* |
| | | "système de santé" ET résilien* ET évalu* ET (cadre OU outil* OU méthode OU approche) ; texte intégral, 10 dernières années, revues, santé publique.<br>*Exclues les analyses de livres et Bibliographies* |
| SSRN | Peer reviewed | "Health system" resilience |
| Google Scholar | Peer reviewed | allintitle: "Health system" resilience; *2012-2022* |
| DOAJ | Peer reviewed | Health system resilience |
| MedRxiv | Preprint | Health system resilience |
| OAIster | Grey literature | ti :"health system" resilience; *Articles et documents d'archives, 2012-2022* |
| ReliefW eb | Grey literature | resilience, "health system" |
| WHO library | Grey literature | "Health system", assessment |

Table S3: Studies assessing health system resilience and countries of implementation

| N° Authors | Countries |
|---|---|
| 1 Alami *et al.*, 2021 | Canada |
| 2 Ammar *et al.*, 2016 | Lebanon |
| 3 Bigoni *et al.*, 2022 | Brazil |
| 4 Burau *et al.*, 2022 | Austria, Denmark, Germany, Italy, Netherlands, United Kingdom |
| 5 Crowe *et al.*, 2014 | United Kingdom |
| 6 Fitter *et al.*, 2017 | Haïti |
| 7 Giarelli, 2020 | China, Italy, South Korea, United Kingdom, United States of America |
| 8 Gilson *et al.*, 2020 | South Africa |
| 9 Haldane *et al.*, 2021 | Argentina, Brazil, China, Egypt, Fiji, Germany, India, Japan, Liberia, Mexico, Mozambique, New Zealand, Niger, Nigeria, Pakistan, Peru, Russia, Singapore |
| | South Korea, Spain, Sri Lanka, Sweden, Thailand, Uganda, United Kingdom, United States of America, Uruguay, Vietnam |
| 10 Kagwanja *et al.*, 2020 | Kenya |
| 11 Karamagi *et al.*, 2022 | Algeria, Angola, Benin, Botswana, Burkina Faso, Burundi, Cabo Verde, Cameroon, Central African Republic, Chad, Comoros, Congo, Congo (Dem. Rep.), Ivory Coast, Equatorial Guinea, Eritrea, Eswatini, Ethiopia, Gabon, Gambia (The), Ghana, Guinea, Guinea-Bissau, Kenya, Lesotho, Liberia, Madagascar, Malawi, Mali, Mauritania, Mauritius, Mozambique, Namibia, Niger, Nigeria, Rwanda, Sao Tome and Principe, Senegal, Seychelles, Sierra Leone, South Africa, South Sudan, Tanzania (United Republic of), Togo, Uganda, Zambia, Zimbabwe |
| 12 Ling *et al.*, 2017 | Liberia |
| 13 Massuda *et al.*, 2021 | Brazil |
| 14 McKenzie *et al.*, 2016 | Nigeria |
| 15 Odhiambo *et al.*, 2020 | South Sudan |
| 16 Ozen and Tuncay, 2021 | Turkey |
| 17 Popic *et al.*, 2021 | Austria, Belgium, Bosnia and Hergozevina, Bulgaria, Croatia, Cyprus, Czech Republic, Denmark, Estonia, Finland, France, Germany, Greece, Hungary, Israël, Italy, Latvia, Lithuania, Netherlands, Norway, Poland, Romania, Russia, Serbia, Slovakia, Slovenia, Spain, Sweden, Ukaine, United Kingdom |
| 18 Rios *et al.*, 2020 | Puerto Rico |
| 19 Thomas *et al.*, 2013 | Ireland |

| | |
|---|---|
| 20 Wang *et al.*, 2020 | Iran, Japan, South Korea, United Kingdom, United States of America |
| 21 WHO Regional Office for Africa, 2018 | Algeria, Angola, Benin, Botswana, Burkina Faso, Burundi, Cabo Verde, Cameroon, Central African Republic, Chad, Comoros, Congo, Congo (Dem. Rep.), Ivory Coast, Equatorial Guinea, Eritrea, Eswatini, Ethiopia, Gabon, Gambia (The), Ghana, Guinea, Guinea-Bissau, Kenya, Lesotho, Liberia, Madagascar, Malawi, Mali, Mauritania, Mauritius, Mozambique, Namibia, Niger, Nigeria, Rwanda, Sao Tome and Principe, Senegal, Seychelles, Sierra Leone, South Africa, South Sudan, Tanzania (United Republic of), Togo, Uganda, Zambia, Zimbabwe |

Table S4: Key elements from the definition of resilience in relation with the health system

| **Key elements** | **Frequency** | **Percentage** |
|---|---|---|
| Maintain | 19 | 17.1% |
| Respond | 14 | 12.6% |
| Prepare | 12 | 10.8% |
| Absorb | 9 | 8.1% |
| adapt | 9 | 8.1% |
| transform | 8 | 7.2% |
| Re-organise | 7 | 6.3% |
| Learn | 6 | 5.4% |
| Recover | 4 | 3.6% |
| Sustain | 3 | 2.7% |
| Anticipate | 2 | 1.8% |
| Foresee | 2 | 1.8% |
| Reduce vulnerability | 2 | 1.8% |
| Resume | 2 | 1.8% |
| Withstand | 2 | 1.8% |
| Cope | 1 | 0.9% |
| Deal with | 1 | 0.9% |
| Emerge | 1 | 0.9% |
| Endure | 1 | 0.9% |
| Mitigate | 1 | 0.9% |
| Prevent | 1 | 0.9% |
| Protect | 1 | 0.9% |
| Re-organise | 1 | 0.9% |
| Support | 1 | 0.9% |
| Understand | 1 | 0.9% |
| **Total occurrence** | **106** | **100%** |

Table S5: Evolution of key references for the conceptual bases proposed or used

| **Period** | **2012-2015** | | **2016-2019** | | **2020-2022** | |
|---|---|---|---|---|---|---|
| Number of reports | 1 | | 6 | | 24 | |
| Number citations | 1 | | 7 | | 44 | |
| References for conceptual bases | Reference | % | Reference | % | Reference | % |
| | WHO, 2007 | 100% | Kruk *et al.*, 2015 | 42,9% | Kruk *et al.*, 2017 | 11,4% |
| **Period** | **2012-2015** | | **2016-2019** | | **2020-2022** | |
| with their percentage of citations | | | WHO, 2007 | 28,6% | WHO, 2010 | 9,1% |
| | | | Paina *et al.*, 2012 | 14,3% | Blanchet *et al.*, 2017 | 6,8% |
| | | | CDC, 2014 | 14,3% | WHO, 2007 | 6,8% |
| | | | | | Kruk at al., 2015 | 4,5% |
| | | | | | Biddle *et al.*, 2020 | 2,3% |
| | | | | | Briguglio, 1995 | 2,3% |
| | | | | | Bruneau *et al.*, 2003 | 2,3% |
| | | | | | Chamberland-Rowe *et al.*, 2019 | 2,3% |
| | | | | | Fischer, 2010 | 2,3% |
| | | | | | Gilson *et al.*, 2017 | 2,3% |
| | | | | | Hanefeld *et al.*, 2018 | 2,3% |
| | | | | | Hollnagel *et al.*, 2006 | 2,3% |
| | | | | | IHR, 2005 | 2,3% |
| | | | | | Jovanović *et al.*, 2018 | 2,3% |
| | | | | | Kagwanja *et al.*, 2020 | 2,3% |
| | | | | | Kruse *et al.*, 2017 | 2,3% |
| | | | | | Norris *et al.*, 2008 | 2,3% |

| | | | |
|---|---|---|---|
| | | Nuzzo *et al.*, 2019 | 2,3% |
| | | Øien *et al.*, 2012 | 2,3% |
| | | Palayi *et al.*, 2019 | 2,3% |
| | | Patel *et al.*, 2017 | 2,3% |
| | | Rogers, 2011 | 2,3% |
| | | Shahabi *et al.*, 2019 | 2,3% |
| | | Thomas *et al.*, 2020 | 2,3% |
| | | Van Schalkwyk *et al.*, 2020 | 2,3% |
| | | Wendt *et al.*, 2010 | 2,3% |
| | | WHO, 2006 | 2,3% |
| | | WHO, 2015 | 2,3% |
| | | WHO, 2016 | 2,3% |
| | | WHO, 2017 | 2,3% |
| | | WHO, 2018 | 2,3% |

Table S6: Chronology of conceptual bases for the assessment of health system resilience

| **Period** | **2012-2015** | | **2016-2019** | | **2020-2022** | |
|---|---|---|---|---|---|---|
| Reports | 3 | | 7 | | 24 | |
| Total occurrence | 3 | | 8 | | 24 | |
| Conceptual bases* and percentage of occurrence | Conceptual base | % | Conceptual base | % | Conceptual base | % |
| | **Health system building blocks framework** | 33,3% | *Health system building blocks framework* | 25,0% | *Health system building blocks framework* | 8,3% |
| | **Framework for assessing the resilience of health systems in terms of how they have adjusted to economic crisis** | 33,3% | ***Health system resilience framework*** | 37,5% | *Health system resilience framework* | 12,5% |
| | **Stylised health system, akin to an industrial process** | 33,3% | **CDC's 10 Essential Public Health Services (EPHSs) framework (CDC's EPHS)** | 12,5% | **The everyday health system resilience (EHSR) framework** | 8,3% |
| | | | **Complex adaptive systems (CAS) theory** | 12,5% | 13 Resilience-enhancing strategies based on health system core functions and shock cycle stages | 8,3% |
| | | | **Data-driven computational framework** | 12,5% | Briguglio's vulnerability and resilience framework | 4,2% |
| | | | | | Community resilience in health systems | 4,2% |
| | | | | | Conceptual framework for EID preparedness | 4,2% |
| | | | | | Conceptual framework of the dimensions of resilience governance | 4,2% |
| | | | | | Determinants of health system resilience framework | 4,2% |

| Period | 2012-2015 | 2016-2019 | 2020-2022 | |
|---|---|---|---|---|
| | | | Emergency preparedness and response (EPR) and inherent system resilience (ISR) based Health System Resilience Index | 4,2% |
| | | | Health System Resilience Analysis meta Framework | 4,2% |
| | | | Health system resilience analytical framework | 4,2% |
| | | | Health System Resilience Assessment Checklist | 4,2% |
| | | | Input-output-outcome | 4,2% |
| | | | Multi-dimensional Health and Social Care Systems (MHSCS) framework | 4,2% |
| | | | Production process | 4,2% |
| | | | SmartResilience project concept of resilience | 4,2% |
| | | | TISM (Total interpretive structural modelling) | 4,2% |
| | | | WHO framework for measuring the climate resilience of health systems | 4,2% |

* New conceptual bases are in **bold**; those that are maintained across the periods are *italicised*.

Table S7: Assessment approaches in reviewed reports

| Author | Year | Category | Approach |
|---|---|---|---|
| Bayntun *et al.* | 2012 | Proposed | System mapping approach |
| Thomas *et al.* | 2013 | Used | Strategy-based approach |
| Crowe *et al.* | 2014 | Used | System mapping approach |
| Ammar *et al.* | 2016 | Used | System mapping approach |
| McKenzie *et al.* | 2016 | Used | System mapping approach |
| Fitter *et al.* | 2017 | Used | System mapping approach |
| Kruk *et al.* | 2017 | Proposed | Capacity-based approach |
| Ling *et al.* | 2017 | Used | Capacity-based approach |
| WHO Regional Office for Africa | 2018 | Used | Capacity-based approach |
| Lo Sardo *et al.* | 2019 | Proposed | System mapping approach |
| Giarelli | 2020 | Used | Strategy-based approach |
| Gilson *et al.* | 2020 | Used | Strategy-based approach |
| Kagwanja *et al.* | 2020 | Used | Strategy-based approach |
| Rios *et al.* | 2020 | Used | Capacity-based approach |
| Wang *et al.* | 2020 | Used | Capacity-based approach |
| Bhandari and Alonge | 2020 | Proposed | System mapping approach |
| Expert Group HSPA | 2020 | Proposed | System mapping approach |
| Odhiambo *et al.* | 2020 | Used | System mapping approach |
| Thomas *et al.* | 2020 | Proposed | System mapping approach |
| Jovanović *et al.* | 2020 | Proposed | System mapping approach |
| Meyer *et al.* | 2020 | Proposed | System mapping approach |
| Etemadi and Tadayon | 2021 | Proposed | Capacity-based approach |
| Pilevari and Shiva | 2021 | Proposed | Mix of the 3 approaches (i) Health system performance, (ii) Capacity-based approach, (iii)Strategy-based approach |
| Alami *et al.* | 2021 | Used | System mapping approach |
| Haldane *et al.* | 2021 | Used | System mapping approach |
| Massuda *et al.* | 2021 | Used | System mapping approach |
| Ozen and Tuncay | 2021 | Used | System mapping approach |
| Rogers *et al.* | 2021 | Proposed | System mapping approach |
| Popic *et al.* | 2021 | Used | System mapping approach |
| Burau *et al.* | 2022 | Used | Strategy-based approach |
| Karamagi *et al.* | 2022 | Used | Mix of the 2 approaches (i) System mapping approach , (ii) Capacity-based approach |
| Foroughi *et al.* | 2022 | Proposed | Mix of the 3 approaches (i) System mapping approach , (ii) Capacity-based approach, (iii) Strategy-based approach |
| Bigoni *et al.* | 2022 | Used | System mapping approach |
| WHO | 2022 | Proposed | System mapping approach |

Table S8: Assessment approaches used, with related study details

| N° | Report | Framework | Assessment approach | Study design | Method | Data source |
|---|---|---|---|---|---|---|
| 1 | Alami *et al.*, 2021 | Conceptual framework for EID preparedness | System mapping | Case study | Evidence synthesis<br>Mixed methods | Literature review; Expert panel<br>Secondary administrative data |
| 2 | Ammar *et al.*, 2016 | Health system building blocks framework | System mapping | Case study | Evidence synthesis<br>Quantitative | Literature review; MOPH, the UN, WB and NGOs secondary data |
| 3 | Bigoni *et al.*, 2022 | Health system building blocks framework | System mapping | Case study | Quantitative | Information System on Public Health Budgets (SIOPS); National Registry of Health Facilities; CNES database |
| 4 | Burau *et al.*, 2022 | Health system resilience analytical framework | Strategy-based approach | Comparative case study | Mixed methods | Written secondary and primary sources; Expert information |
| 5 | Crowe *et al.*, 2014 | Stylised health system, akin to an industrial process | System mapping | Case study | Quantitative (Modelling) | Routine MoH data |
| 6 | Fitter *et al.*, 2017 | CDC's EPHS framework | System mapping | Case study | Not described | Not described |
| 7 | Giarelli, 2020 | Conceptual framework of the dimensions of resilience governance | Strategy-based approach | Narrative around the dimensions | Not described | Not described |
| 8 | Gilson *et al.*, 2020 | The everyday health system resilience (EHSR) framework | Strategy-based approach | Case study | Mixed methods | Notes from observation; Records from IDIs; Meeting minutes; Secondary data |
| 9 | Haldane *et al.*, 2021 | Determinants of Resilient health systems framework | System mapping | Case study | Mixed methods | Literature review; Semi-structured interviews; National government submissions; Experts |
| 10 | Kagwanja *et al.*, 2020 | The everyday health system resilience (EHSR) framework | Strategy-based approach | Case study | Qualitative | Field experience; Informal observations notes; meetings' notes; Records from IDIs |
| 11 | Karamagi *et al.*, 2022 | EPR and ISR based Health System Resilience Index | System mapping and Capacity-based approach | Comparative case study | Mixed methods | Routine surveillance data; IHR SPAR data; Experts |

| N° | Report | Framework | Assessment approach | Study design | Method | Data source |
|---|---|---|---|---|---|---|
| 12 | Ling *et al.*, 2017 | Resilient health systems framework | Capacity-based approach | Case study | Qualitative | Records from semi-structured IDIs/FGDs |
| 13 | Massuda *et al.*, 2021 | Health system building blocks framework | System mapping | Case study | Evidence synthesis Qualitative | Scientific literature and official documents; experts |
| 14 | McKenzie *et al.*, 2016 | CAS theory; Health system building blocks framework | System mapping | Case study | Mixed methods | Project implementation records and reports |
| 15 | Odhiambo *et al.*, 2020 | Briguglio's vulnerability and resilience framework | System mapping | Case study | Quantitative | National surveys on health indicators, UNOCHA dataset |
| 16 | Ozen and Tuncay, 2021 | Input-output-outcome | System mapping | Case study | Quantitative (Modelling) | Demographic data<br>MoH database |
| 17 | Popic *et al.*, 2021 | Production process | System mapping | Comparative case study | Quantitative (modelling) | The Economist; European Health for All database; Johns Hopkins CSSE; COVID-19 government response tracker |
| 18 | Rios *et al.*, 2020 | Resilient health systems framework | Capacity-based approach | Case study | Qualitative | Records from KII |
| 19 | Thomas *et al.*, 2013 | Framework for assessing the resilience of health systems adjustment to economic crisis | Strategy-based approach | Case study | Mixed methods | Budget and policy documents; Reports on key indicators; Key performance metrics |
| 20 | Wang *et al.*, 2020 | Resilient health systems framework | Capacity-based approach | Comparative case study | Mixed methods | WHO COVID-19 dashboard; SPAR results; GHS index<br>Article and press material |
| 21 | WHO Regional Office for Africa, 2018 | Resilient health systems framework | Capacity-based approach | Comparative case study | Mixed methods | Records from KII |

Table S9: Assessment approaches with their levels of implementation across the health system

| Assessment approach | Level of implementation | | |
|---|---|---|---|
| | Country | Local | Subnational |
| Strategy-based approach | 3 | 2 | 0 |
| Capacity-based approach | 4 | 1 | 0 |
| System mapping approach | 9 | 2 | 3 |
